# Unraveling Abdominal Migraine in Adults: A Comprehensive Narrative Review

**DOI:** 10.7759/cureus.43760

**Published:** 2023-08-19

**Authors:** Naveen Kizhakkayil Tency, Archa Roy, Nithya Krishnakumaran, Anju Maria Thomas

**Affiliations:** 1 Department of Internal Medicine, Government T D Medical College, Alappuzha, IND

**Keywords:** abdominal migraine, adult, functional abdominal pain, management, narrative review, recurrent abdominal pain

## Abstract

Abdominal migraine is a condition characterized by recurrent episodes of abdominal pain accompanied by migraine-associated symptoms, primarily affecting pediatric populations. Its occurrence in adults is often overlooked due to limited literature on adult abdominal migraine. This article provides an overview of the current understanding and management of abdominal migraine in adult populations, including the diagnostic criteria, pathophysiology, differentiating features of other associated gastrointestinal pain syndromes, and various treatment approaches based on available literature.

The review acknowledges the limitations, including the scarcity of literature on adult abdominal migraine and the absence of a systematic approach. It emphasizes the need for further research to enhance our understanding of this condition and establish evidence-based treatment guidelines specifically for adults. Accurate diagnosis and patient education are crucial for physicians in recognizing abdominal migraine as a differential diagnosis in cases of long-standing recurrent abdominal pain, promoting the importance of further research to advance our knowledge and improve patient outcomes.

## Introduction and background

Abdominal migraine (AM) a cause of recurrent abdominal pain is a frequently overlooked and underestimated cause of recurring abdominal pain. Although abdominal migraine is frequently observed in children, its prevalence and impact in adults have received limited attention and recognition [1,2]. The phenomenon was first identified in children by Symon and Russel back in 1984 [3]. Diagnosing abdominal migraine can be challenging, as it exhibits symptoms overlapping with various organ systems. However, a correct clinical diagnosis can spare patients from unnecessary treatments and investigations [4]. The International Headache Society's Headache Classification Committee, in their publication The International Classification of Headache Disorders (ICHD), 3rd edition (beta version), [5] and the Rome Foundation, with their Rome 4 criteria [6], have established specific guidelines for the accurate diagnosis of abdominal migraine. These criteria provide a standardized approach to effectively identify and diagnose AM in both children and adults.

Among children, previous studies have reported prevalence rates of abdominal migraine ranging from 5% to 9% [7,8]. However, in adults, the diagnosis of abdominal migraine is very uncommon [4], and only a few case reports have been published to date. The diagnostic challenges arise when a migraine presents only with gastrointestinal symptoms, without accompanying headaches, posing a significant complexity for physicians. This often leads to frustration for patients and medical practitioners due to delays in accurate diagnosis and appropriate treatment, Several case reports of adult abdominal migraine have highlighted the difficulties encountered in clinical practice [9-13].

While it has been widely believed that abdominal migraine is predominantly a childhood disorder that resolves in adulthood, often transitioning into typical migraine headaches [14], there have been rare instances where adults experience both migraine headaches and recurrent abdominal pain [15]. Unfortunately, the lack of case reports and research on abdominal migraine in adults has hindered our understanding of this condition, perpetuating the misconception that it exclusively affects children [12].

To address the knowledge gap surrounding abdominal migraine in adults, this article aims to provide a comprehensive review of the subject. It will cover the definition, prevalence, triggers and relievers, pathophysiology, management, and the association of abdominal migraine with other episodic syndromes and functional gastrointestinal disorders. By consolidating existing knowledge, highlighting clinical features, and addressing diagnostic challenges, this review aims to raise awareness among clinicians and gastroenterologists. The goal is to encourage them to consider abdominal migraine as a potential diagnosis when evaluating adult patients with recurring abdominal pain and migrainous features [5,6,12,16].

By bridging the gap in understanding abdominal migraine in adults, we hope to underscore its significance and prompt healthcare professionals to incorporate this condition into their clinical practice. This, in turn, will lead to improved diagnostic accuracy, reduced unnecessary investigations, and enhanced patient care and outcomes.

## Review

Methodology

We conducted a narrative review to synthesize and connect research on abdominal migraine in adults, utilizing both quantitative and qualitative data [17]. This approach was chosen instead of a systematic review methodology as it allowed us to encompass various methodological and theoretical conceptualizations [18]. The choice to incorporate both quantitative and qualitative methods was made with the intention of gaining a holistic understanding of the subject matter. The purpose of our review was to provide a comprehensive overview of the existing literature on abdominal migraine.

We conducted a comprehensive search in PubMed, Cochrane Library, and Google Scholar using a combination of keywords and Boolean operators. In PubMed and Cochrane Library, we employed Medical Subject Headings (MeSH) terms and free-text keywords. For instance, in PubMed, we used ('abdominal migraine' [Title/Abstract]) AND ('treatment' OR 'management' OR 'pathogenesis' OR 'prevalence' OR 'diagnosis' [Title/Abstract]). We excluded the term 'Adult' as the number of studies focusing specifically on adults was limited. In Google Scholar, we used the search string 'abdominal migraine' adult treatment OR pathogenesis all abstract [Title/Abstract]. The search was limited to English articles or those with English translations. We also employed the snowballing technique by reviewing the reference lists of selected articles to identify additional relevant publications that were not captured in our initial search.

During the screening process, we initially reviewed titles and abstracts to exclude studies that were unrelated to our research objectives. We included peer-reviewed research articles that provided insights into the prevalence, diagnosis, treatment, pathogenesis, and management of abdominal migraine in adults. We aimed to consolidate the existing literature on abdominal migraine rather than document every conceivable study conducted on the topic. We carefully selected articles based on their relevance to our study objectives and their contribution to the overall understanding of abdominal migraine in adults. Any disagreement during the review process was resolved after discussion.

Review

Definition

Abdominal migraine is a distinct entity characterized by recurrent episodes of severe abdominal pain primarily seen in children (ICHD-3). These episodes are paroxysmal and can last for two to 72 hours (ICHD-3) or one hour or more (Rome IV). The pain is typically located in the midline or periumbilical region and has a dull or "just sore" quality with a moderate to severe intensity (ICHD-3). It is associated with vasomotor symptoms such as pallor, anorexia, nausea, and vomiting (ICHD-3; Rome IV). Importantly, as per ICHD-3, during these episodes, there is a complete absence of headache, when a headache or head pain during attacks is identified, a diagnosis of migraine without aura should be considered. Most children with abdominal migraine will develop migraine headaches later in life.

To diagnose abdominal migraine, specific criteria need to be met. Table 1 shows a comparison of the diagnostic criteria provided by ICHD-3 and Rome IV [5,6].

**Table 1:** Comparison of Diagnostic Criteria for Abdominal Migraine: ICHD-3 vs. Rome IV ICHD: International Classification of Headache Disorders [5,6]

Criteria	ICHD-3: 1.6.1.2	Rome IV: H2c
Duration of pain	2-72 hours	One hour or more
Number of attacks	At least five attacks	At least two episodes
Location of pain	Midline, periumbilical, or poorly localized	Periumbilical, midline, or diffuse
Pain characteristics	Dull or "just sore" quality	N/A
Associated symptoms	Anorexia, nausea, vomiting, pallor (at least 2 out of 4)	Anorexia, nausea, vomiting, headache, photophobia, pallor
Symptoms between attacks	Complete freedom from symptoms	N/A
Other conditions ruled out	Gastrointestinal or renal diseases	Other medical conditions
Duration of evaluation	N/A	At least six months before diagnosis

It is worth noting that while both criteria share similarities in terms of abdominal pain and associated symptoms, there are differences in specific details and emphasis. Clinicians and researchers should consider these variations when applying the criteria in practice or conducting further studies.

Prevalence

Abdominal migraine is a recurrent abdominal pain disorder that is commonly associated with children but has been largely neglected in adults [1,2]. The prevalence among children has been reported as high as 9.2% based on a study using Rome III criteria [7]. Furthermore, other studies have shown that abdominal migraine affects approximately 5% to 9% of the pediatric population, with higher rates observed in children with a family history of migraine or depression [8,19]. Additionally, it appears to be more prevalent among girls, with a female-to-male ratio of around 1.6:1 according to Abu-Arafeh's initial prevalence study in 1995 [8]. Despite these findings, the prevalence and characteristics of abdominal migraine in adults have not been extensively studied.

Pathophysiology

**Figure 1:**
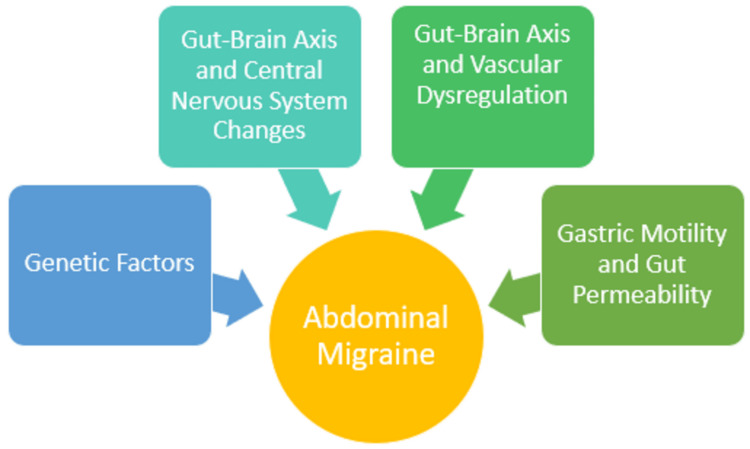
Pathophysiology of Abdominal Migraine: A Flowchart Illustrating the Four Major Mechanisms Image Credit: Naveen Kizhakkayil Tency (Corresponding Author)

Abdominal migraine shares some common pathophysiological features with migraine and cyclic vomiting syndrome (CVS), indicating potential underlying mechanisms. The pathogenesis of abdominal migraine involves specific changes to the gut-brain axis, vascular dysregulation, central nervous system alterations, gastric motility and permeability, and genetic factors in both childhood and adulthood (Figure 1) [20].

**Figure 2:**
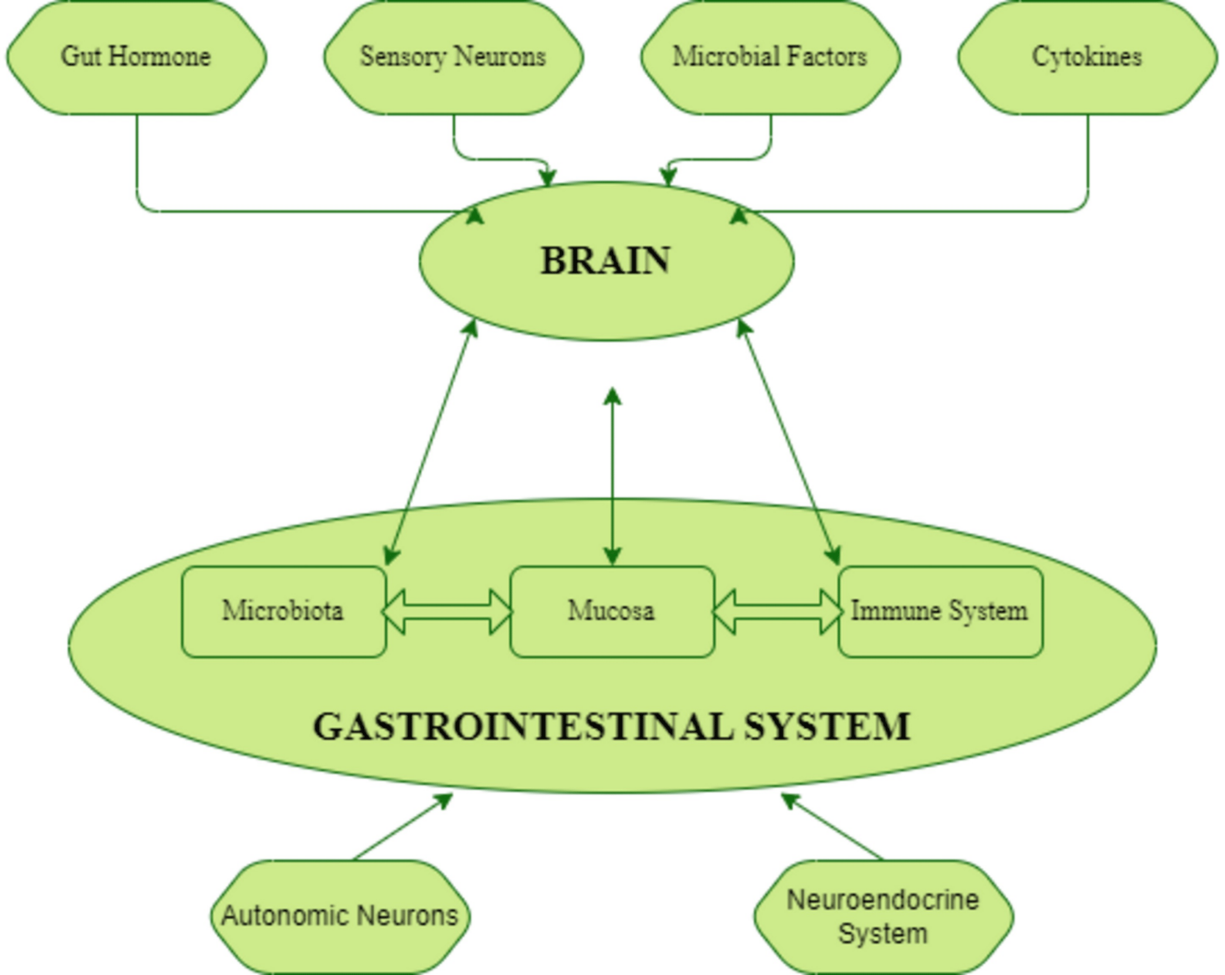
Flowchart Demonstrating Gut-Brain Axis: Bidirectional Interactions and Factors Influencing Cerebral Function and Gut Behavior Image Credits: Naveen Kizhakkayil Tency (Corresponding Author)

Brain-gut axis refers to the exchange of messages between the brain and various components of the gastrointestinal system, including the gut microbiota, mucosa, and immune system. This communication occurs in both directions, with signals traveling from the brain to the gut and vice versa. Additionally, the gut microbiota interacts with the mucosa and the immune system in the gut. Various factors, such as microbial elements, gut hormones, sensory neurons, and cytokines, can influence cerebral function. Conversely, autonomic neurons and neuroendocrine factors can modify gut behavior in response (Figure 2) [4].

Gut-brain axis and vascular dysregulation*: *There is an interaction between the central nervous system and the richly innervated gut in abdominal migraine. The trigeminovascular system, known for its role in migraine headaches, may also play a major role in abdominal migraine [21,22]. While vasospasm of small gut vessels is not suggested as the cause of periumbilical pain, regional or central changes in blood flow are thought to be important, similar to other forms of migraine [21,23,24].

Genetic factors: The strong familial incidence of abdominal migraine suggests a significant genetic role, particularly mutations involving cell membrane transport (channelopathies) [25-27].

Gut-brain relationship and central nervous system changes: The enteric nervous system (ENS), a subdivision of the autonomic nervous system, controls gastrointestinal behavior with little input from the brain or spinal cord [28]. Disruption in the gut-brain axis and communication between the enteric and central nervous systems may contribute to the association between migrainous features and abdominal symptoms [4,20,28].

Gastric motility and gut permeability: Gastric motility and gut permeability have been studied in relation to abdominal migraine. Devanarayana et al. (2016) [29] found significantly lower gastric and antral motility parameters in children with abdominal migraine, with a correlation between symptom severity and gastric motility. Bentley et al. [19] demonstrated increased mucosal permeability of the small intestine in abdominal migraine patients, which showed improvement along with symptom relief. These findings suggest that disturbances in gastric motility and gut permeability may contribute to the pathophysiology of abdominal migraine, highlighting the complex interplay between the gut and the central nervous system.

Triggers and Relief Strategies

Abdominal migraine can be triggered by various factors such as stress, tiredness, travel, missed meals, and changes in routine. These triggers are similar to those observed in other types of migraines [8,30]. However, it is important to note that sometimes these triggers may be mistaken for premonitory symptoms, which are symptoms that precede or forewarn of a migraine episode [5]. An example of this is when bright light or low mood seems to trigger an episode of abdominal migraine, but in reality, these are recognized as premonitory symptoms such as photophobia and mood changes [31].

Similar to other migraines, rest, sleep, and analgesia are common methods for relieving abdominal migraine symptoms [8]. Although initially described primarily in children, subsequent case reports have revealed that the triggers and relief strategies for abdominal migraine are commonly experienced by both children and adults, highlighting the shared nature of these findings across different age groups [9-14].

Gastrointestinal Episodic Syndrome

The ICHD-3 recognizes various episodic syndromes associated with migraine. These syndromes can occur in individuals with migraine without aura or migraine with aura, and they may also manifest in adults. One group of syndromes associated with migraine is recurrent gastrointestinal disturbances (1.6.1). Two prominent conditions within this group are abdominal migraine and cyclic vomiting syndrome. Infantile colic is also sometimes described as a GI disturbance related to migraines (Table 2).

**Table 2:** Differentiating Features of Recurrent Gastrointestinal Episodic Syndrome Associated with Migraine CVS: cyclic vomiting syndrome, CNS: central nervous system, ADHD: attention-deficit/hyperactivity disorder

	Abdominal Migraine	Cyclic Vomiting Syndrome	Infantile Colic
Definition [5]	Episodic central abdominal pain in children; is associated with symptoms like pallor, anorexia, nausea, vomiting, photophobia, and headache	Recurrent episodes of intense nausea and vomiting, typically stereotypical and predictable; complete resolution of symptoms between attacks	Recurrent episodes of irritability, fussing, or crying in infants; episodes last for 3 hours per day and occur on 3 days per week for 3 weeks
Pathophysiology	Involves alterations in gastric motility, gut permeability, and gut-brain axis communication [20]	Mitochondrial abnormalities, altered brain connectivity, autonomic dysfunction, and altered gastric motility [32]	Influenced by microbiota composition, inflammation in the intestinal tract, and CNS immaturity [33,34]
Prognosis	Children with abdominal migraine have an excellent prognosis, with a majority experiencing complete resolution of symptoms [20]	CVS symptoms tend to improve or resolve as children mature; adults may experience longer and more intense episodes [35,36]	Infantile colic may be associated with an increased risk of sleep disorders, aggression, ADHD, recurrent abdominal pain, and migraine [37]
Treatment	Migraine treatments are often used, such as pizotifen, propranolol, cyproheptadine, flunarizine, and lifestyle modifications [20]	Similar treatments as migraine, including lifestyle modifications and medications like propranolol, flunarizine, mirtazapine, and antiemetics [38]	Moderate evidence for the efficacy of Lactobacillus reuteri DSM 17 938 in the treatment of infantile colic; dietary modification may not be effective [39]
Temporal Association with Migraine [4]	Precursor of migraine in adulthood	Precursor of migraine in adulthood	Precursor of migraine in adulthood

In addition to these gastrointestinal disturbances, there are other episodic syndromes associated with migraine. Benign paroxysmal vertigo (1.6.2) is characterized by recurrent brief attacks of vertigo without warning, primarily occurring in healthy children. Benign paroxysmal torticollis (1.6.3) presents as recurrent episodes of head tilt to one side, with or without slight rotation, which spontaneously remits. Since they are not involving the gastrointestinal system, we are not discussing them any further.

In summary, incorporating the discussion of these related conditions CVS and infantile colic when considering abdominal migraines is essential for a comprehensive understanding of the spectrum of migraine-related disorders in pediatric patients.

Functional Abdominal Pain Associated With Migraine

According to the Rome IV classification, functional gastrointestinal disorders (FGID) in children are divided into three subtypes: functional abdominal pain disorders, functional nausea and vomiting disorders, and functional defecation disorders [6]. Functional abdominal pain disorders include three subtypes: irritable bowel syndrome (IBS), functional dyspepsia (FD), and AM (Table 3). It is important to note that IBS and FD are not considered abdominal variants of migraine-like AM, as they coexist with migraine without being temporally linked to it [40].

**Table 3:** Differentiating Features of Functional Abdominal Pain Associated with Migraine

	Irritable Bowel Syndrome (IBS)	Functional Dyspepsia (FD)	Abdominal Migraine
Definition [6]	Abdominal pain is associated with changes in stool frequency and form.	Symptoms include postprandial fullness, early satiation, and epigastric pain.	Recurring episodes of moderate to severe abdominal pain, nausea, vomiting, and loss of appetite.
Pathophysiology	Multifactorial involving biological, psychological, and social factors [41,42]	Involves gastric motility abnormalities, impaired gastric accommodation, and visceral hypersensitivity [43,44]	Involves alterations in gastric motility, gut permeability, and gut-brain axis communication [20]
Temporal Association with Migraine [4]	Co-occurrence with migraine	Co-occurrence with migraine	Precursor of migraine in adulthood
Treatment Options	Parental education, dietary changes, behavioral therapy, and probiotics [45,46]	Limited evidence for pharmaceutical interventions [47]	Migraine treatments are often used, such as pizotifen, propranolol, cyproheptadine, flunarizine, and lifestyle modifications [20]
Psychosocial Factors	Impact of psychosocial factors and psychiatric disorders such as anxiety and depression [41]	Psychosocial factors and psychiatric disorders play a significant role [43]	It is associated with factors such as anxiety, depression, psychosocial difficulties, and abuse. However, the association does not imply causality [48,49]

When discussing AM, it is essential to consider other functional abdominal pain syndromes such as inflammatory bowel disease (IBD) and FD. This is because these conditions present similar symptoms, making it necessary to differentiate between them to provide an accurate diagnosis. Additionally, these syndromes can co-occur with abdominal migraines, further highlighting the need to consider them. Moreover, there is a shared underlying mechanism involving the gut-brain axis, visceral motility, and hypersensitivity, emphasizing the interconnectedness of these conditions. By acknowledging and considering other functional abdominal pain syndromes, healthcare providers can provide comprehensive care that addresses the complexity of symptoms and ensures appropriate management for the patient.

Management

The management of abdominal migraine is crucial in alleviating the recurrent and severe abdominal pain experienced by affected individuals. As the symptoms of abdominal migraine closely resemble those of other gastrointestinal disorders, accurate diagnosis and appropriate treatment are essential for effective management. This section aims to explore the various treatment options reported in the literature, including prophylactic medications, abortive therapies, and lifestyle modifications, to provide insights into the successful management strategies employed for abdominal migraine. The available evidence for adult abdominal migraine mainly comes from case reports [20]. Table 4 presents a comprehensive summary of the documented case reports on adult abdominal migraine, highlighting the management approaches implemented in each case.

**Table 4:** Case Reports and Management Approaches for Adult Abdominal Migraine

Case Report	Age/Sex	Symptoms	Management	Outcome
Kunishi Y et al. (2016) [10]	52-year-old female	One month of recurrent, severe abdominal pain	Loxoprofen (abortive) and lomerizine (prophylaxis)	Relieved
Diao S et al. (2022) [11]	37-year-old male	Seven years of periodic upper abdominal pain, nausea, and vomiting for 2 years.	- Amitriptyline 6.25mg once a night, add 6.25mg every other night if not effective, increased to 50mg during follow-up after 2 months. -Indomethacin suppository or oral ibuprofen and oral domperidone as needed during the acute attack period	The frequency of abdominal pain and nausea attacks decreased (once in 1 to 3 weeks), and the severity of symptoms was significantly reduced
Ibrahim M et al. (2023) [12]	47-year-old Caucasian female	Six months of abdominal pain and vomiting	As-needed sumatriptan (50 mg)	Symptoms were aided and became less frequent over the next three months
D'Onofrio F et al. (2006) [13]	23-year-old female	Recurrent abdominal pain, migraine, and nausea since adolescence	Pizotifen (4-month treatment period)	Complete remission
Woodruff AE et al. (2013) [50]	32-year-old African American female	Recurrent, severe abdominal pain, family history of migraine	Topiramate (prophylactic therapy, 50mg twice daily)	Relieved
Hamed SA (2010) [51]	20-year-old male	Ten years of frequent and prolonged attacks of abdominal colic associated with autonomic manifestations	Valproate (1000mg/day) (prophylactic) eletriptan 40mg per needed) (Abortive)	Improved evoked potential results
Roberts JE et al. (2012) [14]	48-year-old female 24-year-old female	Two and 5 years of symptoms duration respectively that met Rome 3 criteria	Rizatriptan (abortive and topiramate (prophylaxis)	N/A

Literature-Based Approach to Managing Abdominal Migraine in Pediatrics

General management: It is essential to establish a clear diagnosis and provide a comprehensive explanation of abdominal migraine to patients and their families. Educating patients and families about trigger avoidance and maintaining a regular lifestyle is crucial for effective management [8,30]. It is important to avoid using terms like "medically unexplained" or "psychogenic pain" as labeling abdominal migraine in such a manner can exacerbate depression and anxiety in both the child and the parents [20].

Acute/abortive treatment: During abdominal migraine episodes, it is recommended to rest in a dark and quiet environment. Simple analgesics such as paracetamol (15mg/kg) and ibuprofen (10mg/kg) can be used to alleviate pain [8]. A study conducted by Kakisaka et al. on Japanese girls with abdominal migraine found that intranasal sumatriptan (10mg), a serotonin agonist, was effective in managing acute cases [52].

Preventive treatment: Preventive treatment should be considered if there are more than two attacks per month or if the condition significantly impacts the patient's life despite acute treatment [20]. A randomized controlled trial demonstrated that pizotifen, a serotonin agonist, effectively reduces the duration and severity of abdominal migraine when used as a preventive treatment [53]. Pizotifen can be used both as an abortive and prophylactic measure. Retrospective data [54-57] suggest that propranolol (a β-blocker), cyproheptadine (an antihistamine), and flunarizine (a calcium channel blocker) may reduce the frequency and severity of abdominal migraine episodes as preventive therapies. However, it remains unclear whether the evidence from managing childhood migraine headaches can be directly applied to abdominal migraine. Lifestyle modifications are also recommended as a preventive measure to reduce the occurrence of migraine episodes. Table 5 provides a summary of the treatment modalities found in the literature for the preventive treatment of abdominal migraine [20].

**Table 5:** Summary of Treatment Modalities for Preventive Management of Abdominal Migraine

Study Details	Treatment	Adverse Events	Efficacy
Randomized, double-blind, crossover trial [53]	Pizotifen (serotonin agonist) 0.25mg twice daily	Mild drowsiness, weight gain	Improvement in days affected and severity index
Retrospective clinic case note review [54]	Propranolol (Beta Blocker) 10-20mg 2-3 times daily	Drowsiness, weight gain	Excellent response in 75% of patients
Retrospective clinic case note review [54]	Cyproheptadine (antihistamine) 0.25 -0.5mg/kg daily	Drowsiness, weight gain	Excellent response in 33% of patients
Uncontrolled trial [55]	Flunarizine (calcium channel blocker) 5-7.5mg/kg	Not specified	Significant reduction in frequency and duration
Case study [56]	Sodium Valproate (antiepileptic) 500mg 3 times daily IV	Weight gain	Resolved episodes
Case series [57]	Dihydroergotamine (ergot) 0.5mg IV	No significant adverse events	83% responded

Limitations

This narrative literature review has certain limitations that should be acknowledged. Firstly, there is a scarcity of available literature specifically focused on adult abdominal migraine, which may restrict the comprehensiveness of the findings. Additionally, the review's narrative approach, lacking a systematic methodology, may introduce subjectivity and bias into the selection and analysis of the included studies. Furthermore, the absence of an assessment for publication biases raises the possibility of selective reporting. Moreover, the limited number of randomized controlled trials (RCTs) in the literature on adult abdominal migraine treatment poses a challenge in establishing strong evidence-based recommendations. These limitations highlight the importance of conducting further research, including well-designed studies and systematic reviews, to enhance our understanding of the management of abdominal migraine especially in adult populations. Future research endeavors should aim to address these limitations and provide more robust evidence to guide clinical decision-making and optimize patient care.

## Conclusions

In conclusion, abdominal migraine is a unique condition primarily seen in pediatric populations but often overlooked in adults due to a lack of reports. This review has highlighted the importance of accurate diagnosis, patient education, and trigger avoidance. Acute treatment involves rest and simple analgesics, while the review of various case reports suggests the potential effectiveness of various medications including pizotifen, lomerizine, amitriptyline, triptans, topiramate, valproate, and others for preventive treatment in adults. The review acknowledges gaps in knowledge such as a scarcity of literature on the adult population and the absence of a systematic approach in its management. Nonetheless, this underscores the importance for physicians to consider abdominal migraine as a potential differential diagnosis for chronic recurrent abdominal pain. Recommendations for future research include identifying diagnostic criteria specifically for adults, determining its correlation with other functional disorders, and conducting control trials for implementing proper treatment guidelines.
