# Correction: Unraveling Abdominal Migraine in Adults: A Comprehensive Narrative Review

**DOI:** 10.7759/cureus.c415

**Published:** 2026-03-19

**Authors:** Naveen Kizhakkayil Tency, Archa Roy, Nithya Krishnakumaran, Anju Maria Thomas

**Affiliations:** 1 Department of Internal Medicine, Government T D Medical College, Alappuzha, IND

Due to a technical error during the editing process, reference 51 was accidentally changed to list an unrelated and incorrect article. This has been fixed and reference 51 is now correctly listed as below, matching the citation in the text:

Hamed SA: https://dx.doi.org/10.1186/1471-2377-10-2 A migraine variant with abdominal colic and Alice in Wonderland syndrome: a case report and review. BMC Neurol. 2010, 10:2. https://dx.doi.org/10.1186/1471-2377-10-2 10.1186/1471-2377-10-2

